# The urotensin II receptor antagonist, urantide, protects against atherosclerosis in rats

**DOI:** 10.3892/etm.2013.1052

**Published:** 2013-04-09

**Authors:** JUAN ZHAO, QUAN-XIN YU, WEI KONG, HAI-CHENG GAO, BO SUN, YA-QIN XIE, LI-QUN REN

**Affiliations:** 1 Department of Pathophysiology, Chengde Medical University, Chengde, Hebei 067000;; 2 School of Pharmaceutical Sciences, Jilin University, Changchun, Jilin 130021, P.R. China

**Keywords:** artherosclerosis, urotensin II, G protein-coupled receptor 14, urantide, vascular smooth muscle cells

## Abstract

The aim of this study was to explore the use of urantide as an antagonist of the urotensin II (UII) receptor, G protein-coupled receptor 14 (GPR14), to protect against atherosclerosis (AS) in rats. The AS rat model was induced by an intraperitoneal injection of vitamin D3 (VD3) into rats fed with a high-fat diet for four weeks. Urantide was then injected into the rats. Immunohistochemical staining, serum biochemical assay, reverse transcription-polymerase chain reaction (RT-PCR) and western blotting were used to investigate the expression of UII and its receptor GPR14 in the AS rat model. Four weeks after induction, pathological changes typical of AS were observed in the AS rat model. In the plaques of the aortic tunica intima and tunica media, expression of UII and GPR14 was observed. The protein and gene expression levels of UII and GPR14 in the model group were significantly increased compared with those in the normal group (P<0.01). Urantide ameliorated the pathological changes of AS in the rat model and reduced the gene and protein expression levels of UII and GPR14 (P<0.05 or P<0.01). UII is associated with AS and the UII receptor GPR14-specific antagonist, urantide, demonstrates the ability to protect against AS. Thus, this study provides new insight and experimental theories for the clinical application of urantide to treat AS.

## Introduction

Atherosclerosis (AS) is a common disease that is seriously detrimental to human health. Therefore, its mechanism and related treatment methods are areas of interest in medical research. Urotensin II (UII) is the most potent vosoactive cyclic peptide (1,2). By binding to its receptor, G protein-coupled receptor 14 (GPR14), it exhibits biological, hemodynamic and non-hemodynamic effects, as well as apparently pathophysiologic effects on the formation and development of a number of diseases. A large number of fundamental and clinical studies have indicated that UII is closely related to the formation and development of AS (3–5). Thus, the UII receptor antagonist, urantide, may have potential clinical value in the treatment of AS.

Urantide is a peptide similar to human UII and is one of the most potent UII receptor antagonists. Its antagonistic effect is 50 times stronger than that of other chemical compounds (6,7). However, the effect of urantide on AS remains unknown. The present study used a rat model of AS to examine the expression of UII and its receptor GPR14 in rat aorta pectoralis, aiming to determine the effect of urantide on the expression of UII and GPR14 in atherosclerotic rats, thus providing an experimental basis for the clinical prevention of AS.

## Materials and methods

### Reagents

Urantide was synthesized by Shanghai Huadatianyuan Biology Technology Ltd., Co. (Shanghai, China). Fluvastatin (40 mg/box) was purchased from Beijing Nuohua Pharmacy Ltd., Co. (Beijing, China). Dulbecco’s modified Eagle’s medium (DMEM) and UII were purchased from Gibco (Carlsbad, CA, USA); fetal bovine serum (FBS) was purchased from Tianjin Jingyang Corporation (China); α-smooth muscle actin (SMA) was purchased from Beijing Beaosen Biology Technology Ltd., Co. (China); the UII polyclonal and GPR14 polyclonal antibodies were purchased from Santa Cruz Biotechnology, Inc. (Santa Cruz, CA, USA); biotin-labeled IgG was purchased from Wuhan Boster Biological Engineering Co., Ltd. (China); and S-ABC chemical reagent and 3,3′-diaminobenzidine (DAB) developer kits were purchased from Fuzhou Maixin Biotechnology Development Co., Ltd. (China)

### Animals and modeling

A total of 160 healthy male Wistar rats (180–200 g body weight) were provided by the Lab Animal Center of Jilin University (license number: SCXK[JI]-2009-0004). The rats were randomized into two groups: i) the normal group: 20 rats fed on common forage; and ii) the model group: 140 rats fed on a high fat diet and injected with 70 U/kg vitamin D3 (VD3) for three continuous days. The ingredients of the high fat diet included common forage, 3.5% cholesterol, 10% hog fat, 0.2% propylthiouracil, 0.5% sodium cholate and 5% refined sugar. Four weeks later, a hematoxylin and eosin (H&E) staining assay was peformed to observe the morphological changes in the aorta pectoralis in the AS model rats.

Following successful modeling, AS rats were randomly divided into three groups: the model group (20 rats, control), the fluvastatin group (20 rats) and the urantide group (20 rats, divided into three subgroups according to the duration of treatment of 3, 7 and 14 days). The rats in the normal and model groups were intravenously injected with 30 *μ*g/kg normal saline every day. The rats in the fluvastatin group were injected with 5 *μ*g/kg fluvastatin every day for 14 continuous days. The rats in the urantide group were injected with 30 *μ*g/kg urantide once daily and the duration of treatment was 3, 7 and 14 days, respectively. All animal experiments were approved by the Ethics Committee of Jilin University College of Pharmaceutical Sciences (Changchun, China).

### Blood fat and calcium (Ca^2+^) levels of AS rats

Blood samples were collected at the beginning of the experiment, before the injection and at the end of the experiment. Blood collection procedures were as follows: all rats were fasted overnight. Following anesthesia with 0.3% pentobarbital sodium (30 mg/kg), the aorta pectoralis was dissected and arterial blood was collected with a 5-ml injector. Blood serum was separated via 1,500 × g centrifugation for 15 min, allocated to Eppendorf tubes and stored at −20°C.

An automatic biochemistry analyzer was used to examine the levels of triglycerides (TG) in blood serum, as well as total cholesterol (TC), high-density lipoprotein (HDL), low-density lipoprotein (LDL) and Ca^2+^ levels. A hydroxyproline (HYP) kit was used to analyze the concentration of HYP in the blood serum and urine.

### Gene and protein expression levels of UII and GPR14 in the AS rat model

At the end of the experiment (16 weeks), the aorta pectoralis (∼1 cm long) was sampled, placed into a sample bag and immediately frozen in liquid nitrogen at −80°C for storage. Reverse transcription-polymerase chain reaction (RT-PCR) and western blotting were performed to determine the gene and protein expression levels of UII and GPR14 in the AS rat model.

### Statistical analysis

Data are expressed as mean ± standard deviation (SD). Significant differences were examined using SPSS 13.0 software (SPSS, Inc., Chicago, IL, USA). Analysis of variance or rank test were used as the statistical methods to analyze the interclass difference and the least significant difference (LSD) method was used for multiple comparisons of ad hoc test. P<0.05 was considered to indicate a statistically significant result.

## Results

### Textural structure of the aorta pectoralis in the AS rat model

**Figure 1:**
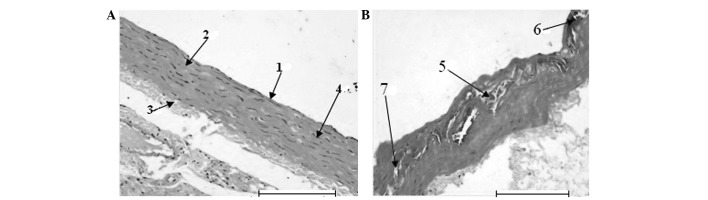
Microscopic aorta pectoralis of rats (H&E staining; magnification, x200; bar indicates 100 *μ*m). (A) normal group; (B) model group. Arrows 1, vascular tunica externa; 2, vascular tunica media; 3, vascular tunica intima; 4, elastic fibers; 5, broken elastic fibers in vascular tunica media in atherosclerotic state; 6, calcification in atherosclerotic state; 7, foam-like cells in atherosclerotic state. H&E, hematoxylin and eosin.

The H&E staining revealed that the blood vessel endothelium in the normal group was integrated, the tunica media had fusiform-shaped smooth muscle cells and the structure of elastic fibers was clear and integrated (Fig. 1A). The tunica intima of the disease region in the model group thickened significantly. The vascular endothelium was not integrated. The tunica media presented apparent hyperplasia, calcification and abundant foam-like accumulation of smooth muscle cells, degeneration, breakage and disintegration of elastic fibers and atrophia, which are typical pathological changes of AS (Fig. 1B). All the results demonstrated that the AS rat model was successfully created by injection with VD3 and feeding with a high-fat diet.

### Effect of urantide on blood fat and Ca^2+^ levels in the AS rat model

At the end of the experiment (16 weeks), the animals were sacrificed. Table I shows that the Ca^2+^, TG, TC, HDL and LDL levels of blood serum in the model group were increased significantly compared with those in the normal group (P<0.01, respectively). However, the Ca^2+^, TG, TC, HDL and LDL levels of blood serum in the fluvastatin group were significantly reduced compared with those in the model group (P<0.01, respectively). Indicators of blood serum in the urantide groups decreased gradually in a dose-dependent manner. Levels in the fluvastatin group were significantly different compared with normal (P<0.01).

### Effect of urantide on HYP levels in the AS rat model

**Figure 2:**
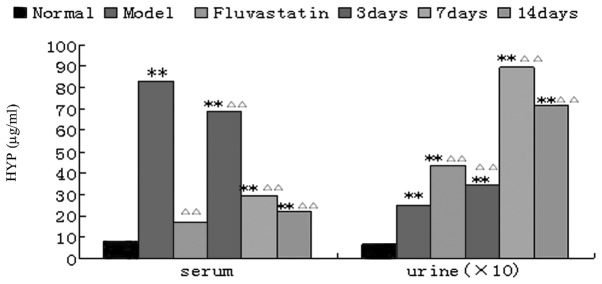
Hydroxyproline (HYP) concentrations in serum and urine in various groups (mean ± standard deviation). ^**^P<0.01 vs. the normal group ^ΔΔ^P<0.01 vs. the model group.

The concentrations of HYP in blood serum and urine in the model group were significantly increased compared with those in the normal group (P<0.01). The HYP level in blood serum in the fluvastatin group was significantly reduced compared with that in the model group (P<0.01). The HYP level in blood serum was significantly lower than that in the model group after injecting urantide for three days (P<0.01), was markedly lower after seven days and even lower after 14 days, and approximate to the level in the fluvastatin group. The urinary HYP levels in the urantide groups and the fluvastatin group were significantly increased (P<0.01) compared with the HYP level in the model group; in the urantide group, the level peaked after 7 days of drug administration (Fig. 2).

### Effect of urantide on mRNA and protein expression of UII and GPR14 in the AS rat model

**Figure 3:**
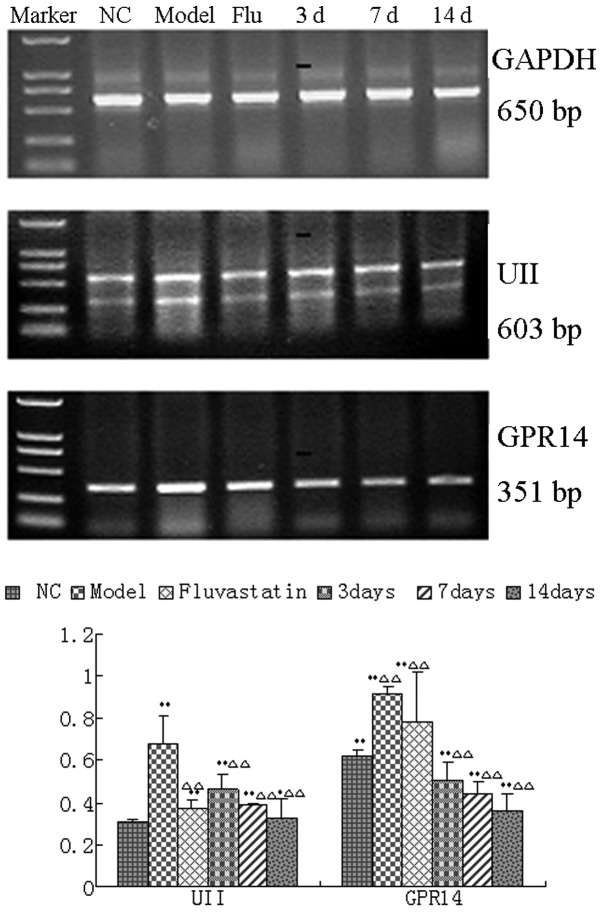
Expression of UII and GPR14 mRNA in the aorta pectoralis (relative grayscale vs. β-actin). NC, the normal group. ^*^P<0.05, ^**^P<0.01 vs. the normal group; ^ΔΔ^P<0.01 vs. the model group. UII, urotensin II; GPR14, G protein-coupled receptor 14; GAPDH, glyceraldehyde 3-phosphate dehydrogenase; Flu, fluvastatin.

The RT-PCR results revealed that UII and GPR14 mRNA expression levels in the aorta pectoralis in the model group were significantly increased compared with those in the normal group (P<0.01). However, the mRNA expression levels in the aorta pectoralis in the urantide groups and the fluvastatin group were significantly reduced compared with those in the model group (P<0.01). The mRNA expression levels in the urantide groups decreased gradually in a dose-dependent manner, reaching the lowest level after 14 days of injection of urantide (Fig. 3).

**Figure 4:**
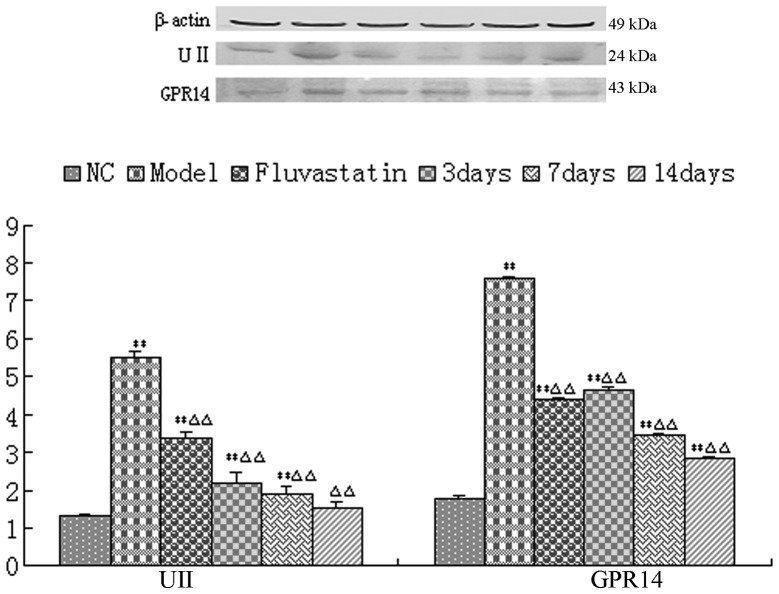
UII and GPR14 protein expression in the aorta pectoralis (relative grayscale vs. β-actin). ^**^P<0.01 vs. the normal (NC) group; ^Δ^P<0.05, ^ΔΔ^P<0.01 vs. the model group. UII, urotensin II; GPR14, G protein-coupled receptor 14.

The western blotting results revealed that the UII and GPR14 protein expression levels in the aorta pectoralis in the model group were significantly increased compared with those in the normal group (P<0.01). However, the protein expression levels in the urantide groups and the fluvastatin group were significantly reduced compared with those in the model group (P<0.01). The protein expression levels in the urantide groups decreased gradually as the injection of urantide continued, reaching the lowest levels after 14 days of injection of urantide (Fig. 4).

## Discussion

The experiments of the present study were designed with referrence to previous reports (8,9), in order to establish a new method for generating the AS model with more serious pathological changes in a short experimental period. We used traditional high-fat diet supplemented with propylthiouracil, sodium cholate and white sugar in order to inhibit thyroid function and stimulate the absorption of cholesterin, as well as overcome the bitter taste of propylthiouracil and increase the blood sugar of rats. Furthermore, we triggered the overload of calcium in the artery via the intraperitoneal injection of 70 U/kg VD3 as the revulsant of calcium ions.

The results of the present study indicated that the supplemented high-fat forage increases the levels of Ca^2+^, TG, TC, HDL and LDL in blood serum in the model group four weeks after feeding. The aortic tunica intima of rats presented typical pathological changes, including the apparent calcification, hyperplasia and foam-like accumulation of vascular smooth muscle cells, the degeneration, breakage and disintegration of elastic fibers and the atrophia of the tunica media. These results demonstrated that the intraperitoneal injection of 70 U/kg VD3 combined with supplemented high-fat forage for four weeks successfully produces a rat model of AS, with a 100% success rate in two separate modeling experiments. Additionally, the survival rate of rats in the AS model group reached 78%. Therefore, the AS models we reproduced in the present study had the advantages of short cycle time, high survival rate and stability compared with other modeling methods (6,9–11).

UII is a vasoactive cyclic peptide originally isolated from the caudal neurosecretory organ of a fish. UII and its receptor GPR14 combine together to form a UII/GPR14 system, thus initiating a series of biological effects, including vasoconstriction (6,10). UII and GPR14 are mainly distributed in the tunica externa of the chest aorta in healthy rats and a small amount is distributed in the tunica intima and tunica media. A certain amount of UII is present in coronal AS plaques, smooth muscle cells with lipidosis and rich areas of macrophages. The vasoconstriction observed in the present study indicated that abundant particles positive for UII and GPR14 expression were present in the aortic tunica intima and tunica media plaque. The RT-PCR and western blotting results further indicated that the gene and protein expression levels of UII and GPR14 were increased in the chest aorta, consistent with previous reports (6,10). Thus, we consider that UII may be directly or indirectly involved in the formation and development of AS.

UII is strongly associated with vasoconstriction; however, the underlying mechanism is complicated. The vasoconstrictive action may be mediated by protein kinase C (PKC), Ca^2+^, phosphatide C, protein kinase and protein tyrosine kinase (PTK) and blocked by PKC inhibitors, Ca^2+^ tunnel antagonists, phosphatidase C inhibitors, PTK inhibitors and kinase inhibitors (7,12,13). Urantide is one of the most potent UII receptor antagonists (3). A previous study demonstrated that urantide competitively antagonizes the pro-contraction effect of UII in the chest aorta of rats and markedly inhibits the increase of UII activity in human monocyte-derived macrophages, thereby blocking the development and accumulation of foam cells (3).

In the present study, the urantide groups were divided into three groups according to the duration of treatment (3, 7 and 14 days) with 30 *μ*g/kg urantide, to observe whether urantide protects against AS and blocks the development of AS. We identified that after three days, urantide resulted in reductions in the concentrations of TG, TC, HDL and LDL in blood serum and reductions in the immunohistochemical intensity and range in the plaques of the aortic tunica intima and tunica media compared with the model group. Urantide also resulted in reductions in the gene and protein expression levels of UII and GPR14. Furthermore, as the injections of urantide continued, the therapeutic effect became more evident. This finding demonstrated that urantide protected the rats from AS, possibly by antagonizing the UII receptor, GPR14.

In conclusion, UII accelerated the initiation and development of AS; this action may be a direct result of the binding of UII to its receptor, GPR14. The gene and protein expression levels of UII and GPR14 in the chest aorta of the AS rats was reduced due to inhibition by treatment with the GPR14-specific antagonist urantide. Urantide inhibits AS and relieves its symptoms, thus providing a theoretical basis for clinical treatment.

## Figures and Tables

**Table I:** Effect of urantide on lipid and calcium concentrations of AS rats at the end of the 16-week experiment (mmol/l).

Group	n	Ca^2+^	TG	TC	HDL	LDL
Normal	10	2.62±0.06	0.03±0.01	1.20±0.02	0.57±0.04	0.16±0.01
Model	10	3.82±0.01^a^	1.74±0.08^a^	17.61±0.08^a^	2.19±0.05^a^	16.05±0.15^a^
Fluvastatin	10	3.12±0.01^a,c^	0.74±0.05^a,c^	12.45±0.02^a,c^	1.27±0.00^a,c^	11.95±0.06^a,c^
Urantide						
3 days	10	3.57±0.00^a,c^	1.04±0.00^a,c^	17.03±0.01^a,c^	1.98±0.00^a,c^	15.16±0.01^a,c^
7 days	10	3.48±0.01^a,c^	0.72±0.14^a,b^	15.87±0.00^a,c^	1.81±0.02^a,c^	14.20±0.01^a,c^
14 days	10	3.43±0.39^a,c^	0.43±0.04^a,c^	12.83±0.06^a,c^	1.48±0.06^a,c^	10.16±0.05^a,c^

Data are presented as mean ± standard deviation.

a P<0.01 vs. the normal group;

b P<0.05,

c P<0.01 vs. the model group. TG, triglycerides; TC, total cholesterol; HDL, high-density lipoprotein; LDL, low-density lipoprotein.
